# NiSe_2_/CdS composite nanoflakes photocatalyst with enhanced activity under visible light

**DOI:** 10.1039/d0ra09272j

**Published:** 2020-11-17

**Authors:** Shijie Shen, Linghui Yan, Kai Song, Zhiping Lin, Zongpeng Wang, Daming Du, Huanhuan Zhang

**Affiliations:** School of Materials Science and Engineering, Lanzhou University of Technology Lanzhou 730050 China; Xuanda Industrial Group Co., Ltd Wenzhou 325105 China; School of Pharmaceutical and Materials Engineering, Taizhou University Taizhou 318000 China shensj@tzc.edu.cn wzmiai@163.com

## Abstract

Degrading organic pollutants using a photocatalyst under visible light is one of the effective ways to solve the increasingly serious environmental pollution problem. In this work, we have loaded a small amount of NiSe_2_ nanoflakes on the surface of CdS using a simple and low-cost solvothermal synthesis method. The samples were characterized with detailed X-ray powder diffraction (XRD), scanning electron microscopy (SEM), transmission electron microscopy (TEM), X-ray photoelectron spectroscopy (XPS), electrochemical impedance spectroscopy (EIS), photocurrent, photoluminescence spectrometer (PL), photocatalytic properties, *etc.* The results show that a 2 mol% load of NiSe_2_ increases the rate of degradation of Rhodamine B (RhB) to more than twice the original rate (0.01000 min^−1^*versus* 0.00478 min^−1^). Meanwhile, the sample has excellent stability. The improved photocatalytic properties can be attributed to the face-to-face contact between the nanoflakes, accelerated separation and transfer of photon-generated carriers. This work provides a suitable co-catalyst that can be used to optimize the performance of other photocatalytic materials.

## Abstract 2

The obtained NiSe_2_/CdS composite nanoflakes exhibit greatly enhanced photocatalytic properties due to the accelerated separation of photon-generated carriers.

## Graphical abstract

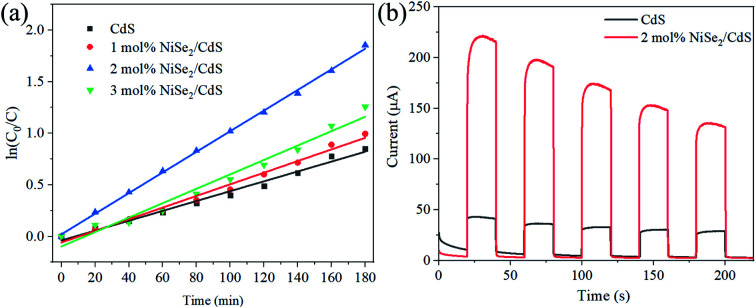

## 1. Introduction

The rapid development of the industrial economy has brought a highly developed material civilization for mankind, but it has also brought about problems of resource reduction and environmental pollution. Therefore, new technologies need to be developed to solve the aforementioned energy and environmental crisis. Solar energy is an inexhaustible renewable energy source. The average hourly solar energy provided to the earth exceeds the global annual energy consumption.^1^ So, converting solar energy into hydrogen energy and degrading pollutants under light are constructive solutions.^2–7^ The realization of the above technologies requires the use of photocatalysts. The earliest developed photocatalytic material was TiO_2_. In 1972 Fujishima and Honda^8^ first reported photocatalytic decomposition of water to produce hydrogen using TiO_2_ single crystals. In the following decades, people have developed a variety of semiconductor photocatalytic materials, such as oxides, sulfides, and oxynitrides.^9–16^ The photocatalytic process involves absorbing sunlight to excite photo-generated carriers, diffusion of carriers, and surface reactions.^17^ The key problems to be solved in the development of efficient photocatalysts include enhancing light absorption and reducing carrier recombination. These problems are related to the crystal structure and band gap of photocatalytic materials. If the band gap is wide, for example, TiO_2_ (3.2 eV) can only absorb ultraviolet light. But ultraviolet light only accounts for 3–5% of the total solar energy,^18^ so the photocatalytic efficiency is severely limited. If the semiconductor has a narrow band gap, it can absorb most of the sunlight. CdS is such a semiconductor (with a band gap of 2.4 eV),^19^ which is suitable as a visible light-responsive photocatalytic material.

In order to improve the photocatalytic performance of CdS, two methods are usually adopted. One of them is to regulate the morphology. Specifically, it includes four types of topography, zero-dimensional (quantum dots), one-dimensional (nanorods), two-dimensional (nanosheets) and three-dimensional structures (hierarchical dendrites).^20–24^ Different types correspond to different mechanisms for optimizing photocatalytic activity.^25^ However, a single semiconductor catalyst still faces the problem of rapid recombination of photogenerated electrons and photogenerated holes. Therefore, a method of compounding with other materials was later developed, specifically including compounding semiconductors (TiO_2_, C_3_N_4_, MoS_2_, *etc.*) and metals (Pt, Au, Ag, *etc.*).^26–33^

NiSe_2_ has two kinds of crystal structures, orthogonal phase and cubic phase, which can be prepared at different temperatures.^34^ Experimental and theoretical results indicate that both phases are metals.^34,35^ The excellent electronic properties of NiSe_2_ make it have a wide range of applications. It was used as the counter electrode of dye-sensitized solar cells and has higher power conversion efficiency than Pt.^36^ As an electrocatalytic hydrogen production catalyst, it has a low overpotential.^37^ It can be used as the anode of the sodium ion battery with a high discharge capacity.^38^ In addition, it can also be used as supercapacitor, oxygen reduction catalyst, thermoelectric material and so on.^39–41^ In view of the electronic properties and rich application potential of NiSe_2_, we speculate that it should be an excellent photocatalytic co-catalyst. However, as far as we know, there is no relevant research so far.

In this work, NiSe_2_ with different loadings were coated on the CdS surface by a simple solvothermal synthesis and their performance of photocatalytic degradation of RhB were investigated under visible light. The results show that it is indeed an excellent co-catalyst. A mere 2% load can increase the degradation rate to more than twice. In addition, the stability of the sample and the mechanism of optimized performance have also been studied.

## 2. Experimental section

### 2.1. Synthesis of NiSe_2_/CdS composite

First, CdS was synthesized in the same way as in the literature.^42^ Then, 2 mmol CdS, 0.04 mmol (0.008 mmol, 0.012 mmol) Se and 0.05 mmol (0.1 mmol, 0.15 mmol) NaBH_4_ were added into 30 mL DMF and stirred for half an hour. Then 0.02 mmol (0.04 mmol, 0.06 mmol) NiCl_2_·6H_2_O was added and stirred for 20 min. The above solution was placed to a 50 mL reactor and reacted at 160 °C for 24 hours. After natural cooling, the powder was washed with ethanol and deionized water, centrifuged, and dried to obtain the desired sample.

### 2.2. Material characterizations

Characterization of samples includes X-ray powder diffraction (XRD, D/MAX 2500), scanning electron microscopy (SEM, S-4800), transmission electron microscopy (TEM, JEM-2100F), X-ray photoelectron spectroscopy (XPS, ESCALAB 250Xi), photoluminescence spectrometer (PL, F-4600).

### 2.3. Photocatalytic reaction

The photocatalytic activity of the sample was characterized by measuring the concentration of RhB under visible light. 50 mg of photocatalyst was added to 50 mL of RhB solution. The solution was stirred for 1.5 hours in the dark until the equilibrium of adsorption and desorption was reached. The light source is a 300 W xenon lamp (cut-off wavelength is 420 nm).

### 2.4. Electrochemical tests

Electrochemical impedance spectroscopy (EIS) and transient photocurrent test were performed in the electrochemical workstation (CHI660C). Specifically, a three-electrode system was used for testing. The working electrode is a piece of ITO coated with the sample. Pt foil is the counter electrode. The standard calomel is the reference electrode. 0.5 M sodium sulfate solution is the electrolyte. The light source is the same with that in the photocatalytic test. The preparation process of the working electrode was as follows: 5 mg photocatalyst was dispersed in 10 microliters Nafion solution (5 wt%) and 1 mL alcohol, and the above solution was sonicated for 1 hour until uniform. Coat 40 microliters of the above solution on a circular ITO surface (5 mm in diameter). Finally, the above ITO was heated at 200 °C for 1 hour in an argon atmosphere.

## 3. Results and discussions

**Fig. 1:**
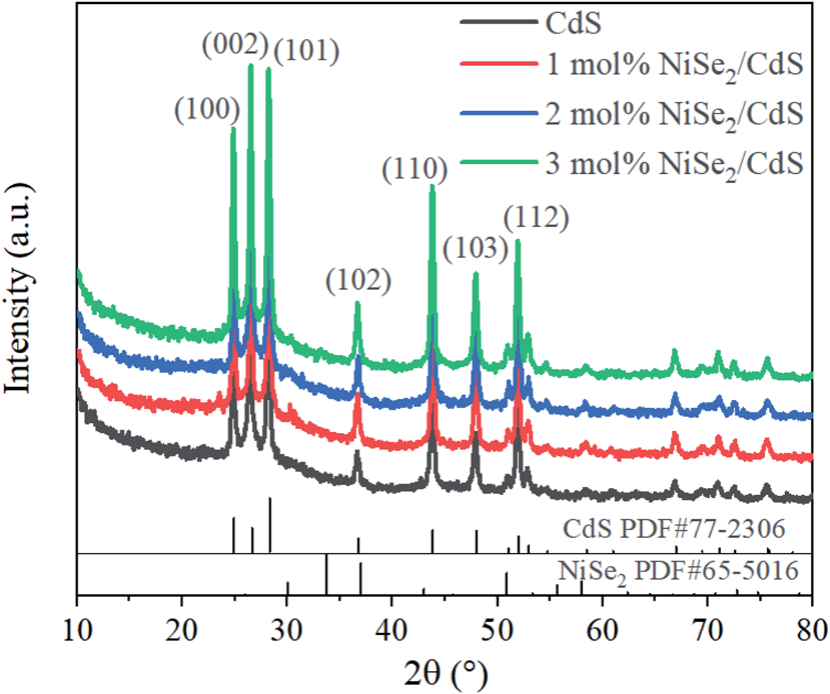
XRD patterns of CdS and 1 mol%, 2 mol%, 3 mol% NiSe_2_ composited CdS.

Fig. 1 shows the XRD patterns of pure CdS and CdS compounded with different molar ratios (1 mol%, 2 mol%, 3 mol%) of NiSe_2_. The main peaks at 24.8°, 26.5°, 28.1°, 36.6°, 43.8°, 47.9°, 51.9° root in lattice planes (100), (002), (101), (102), (110), (103), (112) of CdS (PDF#77-2306), which confirms the products contain CdS and its high purity. With the introduction of NiSe_2_, no additional diffraction peaks appeared. This may be because the amount of Ni is too small to reach the detection level.

**Fig. 2:**
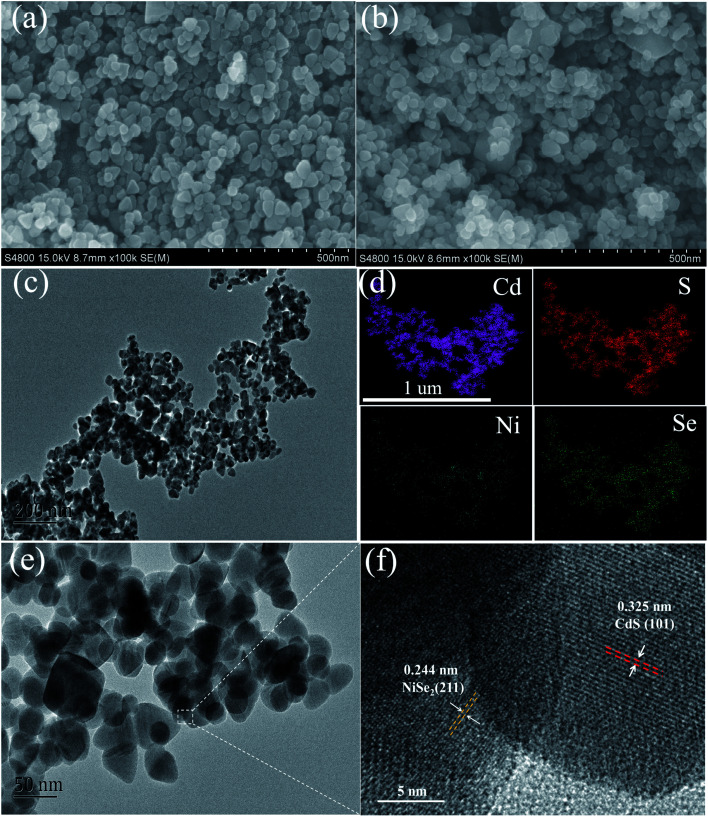
(a) SEM images of CdS, (b–f) SEM images, TEM images, EDS mapping images, high-resolution TEM images of 2 mol% NiSe_2_ composited CdS.

The morphology of the samples is shown in Fig. 2. As for pure CdS, uniform nanoflakes structures can be observed (Fig. 2a). The size of the nanoflakes is about 50 nm and the shape is irregular. After compounding 2 mol% NiSe_2_, there was no significant change in appearance, except for a small number of larger nanoflakes (Fig. 2b). This may be due to the similar morphology of the NiSe_2_ nanoflakes to CdS.^43^ This kind of face-to-face contact between these CdS nanoflakes and NiSe_2_ nanoflakes will help to increase the area of contact, thereby improving the charge transfer between them. The TEM of the sample also shows analogous nanoflakes morphology (Fig. 2c). The EDS mapping confirms the existence of CdS and NiSe_2_, and that these four elements are evenly distributed in the sample (Fig. 2d). Due to the low content of NiSe_2_, the brightness is significantly weaker than CdS. The high-resolution TEM shows two kinds of lattice fringes, corresponding to the (101) crystal plane of CdS and the (211) crystal plane of NiSe_2_. This result confirms the good crystallinity of these two compounds.

**Fig. 3:**
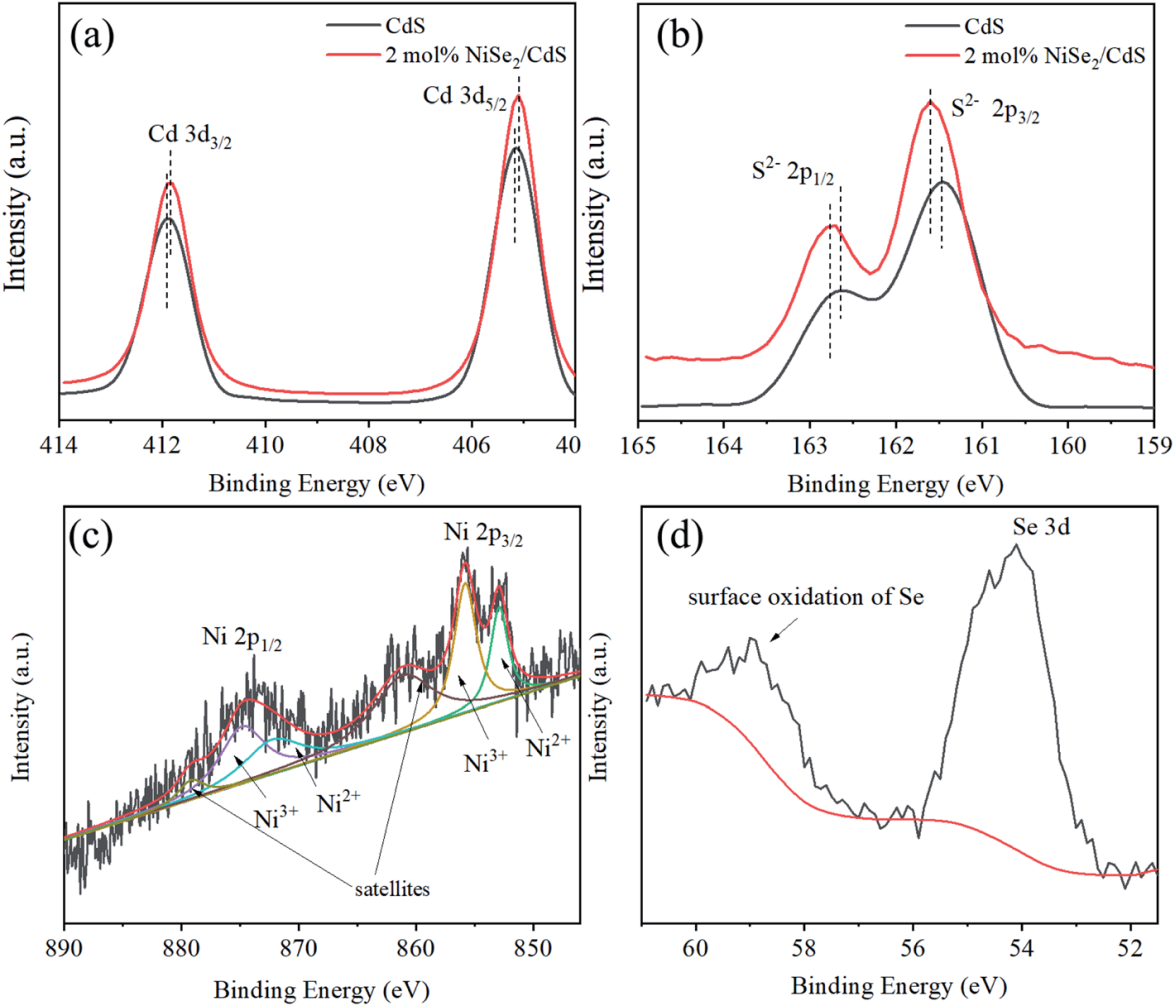
(a) XPS spectra of Cd and (b) XPS spectra of S for CdS and 2 mol% NiSe_2_ composited CdS, (c) XPS spectra of Ni and (d) XPS spectra of Se for 2 mol% NiSe_2_ composited CdS.

The XPS of the samples is shown in Fig. 3. The peaks at 411.9 eV and 405.2 eV correspond to Cd 3d_3/2_ and 3d_5/2_ electrons, respectively (Fig. 3a). The XPS of S can be well fitted with two peaks at 162.7 eV and 161.5 eV, which can be assigned to 2p_1/2_ and 2p_3/2_ electrons of S^2−^ (Fig. 3b).^44^ It can also be seen that after the introduction of NiSe_2_, the peaks of Cd and S have shifted slightly, indicating a charge transfer between CdS and NiSe_2_. As for the XPS of Ni, it is a bit more complicated. The spectrum can be deconvolved to six peaks (Fig. 3c). The peaks at 852.9 eV and 872.2 eV are derived from 2p_3/2_ and 2p_1/2_ electrons of Ni^2+^. The peaks at 855.8 eV and 874.8 eV can be ascribed to 2p_3/2_ and 2p_1/2_ electrons of Ni^3+^, which is due to surface oxidation. The peaks at 861.1 eV and 879.1 eV are the shakeup satellites. These results are similar to those in the literature.^43^ As shown in Fig. 3d, the binding energy signal of Se 3d electrons is situated in 54.1 eV, while the bulge at 59.0 eV is due to the surface oxidation of Se.^43^

**Fig. 4:**
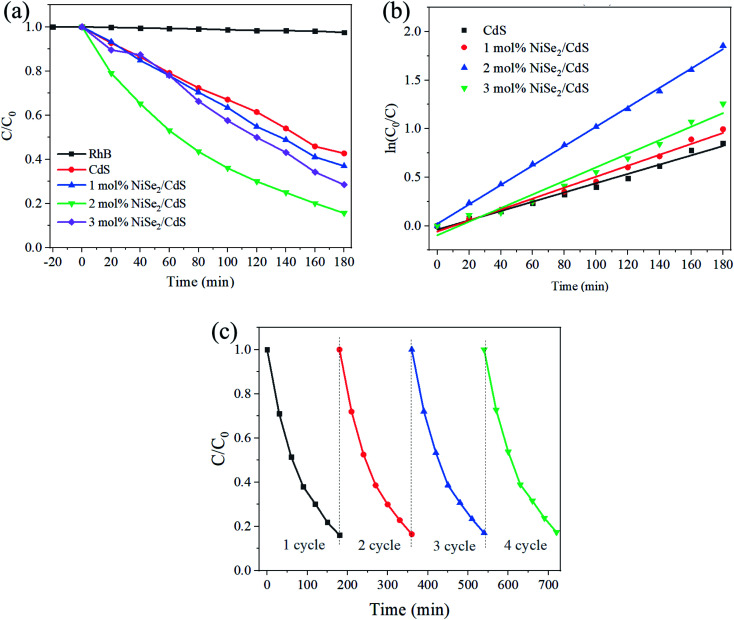
(a) The concentration of RhB under the photocatalytic degradation of different photocatalysts. (b) Degradation rate curve based on the concentration of RhB. (c) Cycle performance of 2 mol% NiSe_2_ composited CdS.

The photocatalytic performance of the samples is shown in Fig. 4. It can be seen that for pure CdS, the concentration of RhB remains 43% after 3 hours of degradation. With the addition of NiSe_2_, the degradation process gradually accelerates. For the added amount of 2 mol%, the degradation is the fastest, with 15% remaining after 3 hours. Continuing to increase NiSe_2_ will cause slower degradation, which may be due to excessive NiSe_2_ blocking a portion of visible light and thus reducing light absorption. The degradation rate is fitted as the kinetics equation: ln(*C*/*C*_0_) = *kt*.^45^ As shown in Fig. 4b, the *k* values are 0.00478 min^−1^, 0.00564 min^−1^, 0.01000 min^−1^, 0.00698 min^−1^ for CdS and 1 mol%, 2 mol%, 3 mol% NiSe_2_ composited CdS. Therefore, the degradation rate has reached more than twice that before compounding. The stability of the sample was also studied. After 4 cycles, the degradation activity did not deteriorate significantly (Fig. 4c), indicating excellent stability.

**Fig. 5:**
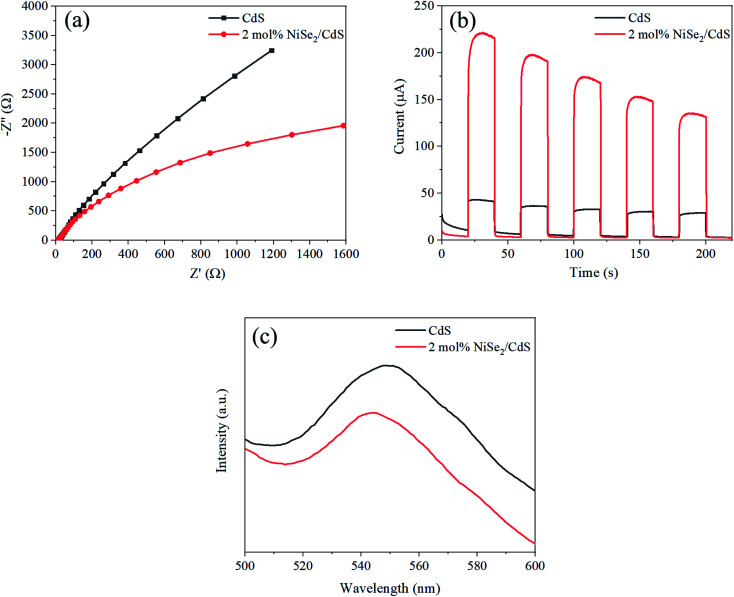
(a) EIS Nyquist plots, (b) transient photocurrent and (c) PL spectrum of CdS and 2 mol% NiSe_2_ composited CdS.

In order to reveal the mechanism of performance enhancement, a series of characterizations were conducted. Electrochemical impedance spectra (EIS) can be used to analyze the resistance of the carriers as they migrate. The radius of the arc is directly related to the resistance. As shown in Fig. 5a, the arc radius of 2 mol% NiSe_2_ composited CdS is significantly smaller than that of CdS, indicating that the former has a smaller charge transfer impedance. The transient photocurrent characterization can reflect the magnitude of the photogenerated current. As Fig. 5b shows, the photocurrent value of 2 mol% NiSe_2_ composited CdS is much larger than that of CdS. This shows that the composite NiSe_2_ can greatly promote the separation and transfer of photogenerated carriers. The PL spectra of the samples are shown in Fig. 5c. The wide bulge near 550 nm is caused by the recombination of photo-generated electrons and photo-generated holes.^46^ The strength of the bulge for the sample with NiSe_2_ was significantly weakened. This shows that NiSe_2_ can indeed promote the separation of photogenerated carriers and reduce recombination.

## 4. Conclusion

We prepared CdS samples uniformly compounded with different contents of NiSe_2_ nanoflakes by solvothermal synthesis. After adding a small amount of NiSe_2_ (2 mol%), the performance of CdS photocatalytic degradation of RhB under visible light has been greatly improved, and the degradation rate has increased by more than 2 times. The reason for the improved activity can be attributed to the fact that NiSe_2_ accelerates the separation and transfer of photogenerated carriers, thereby reducing the occurrence of recombination. The simplicity and low cost of the preparation method and the high activity indicate that NiSe_2_ can be used as an efficient co-catalyst for incorporation into other existing photocatalytic materials.

## Conflicts of interest

There are no conflicts to declare.

## Supplementary Material
